# Growth and Metabolic Response of Chinese Perch to Different Dietary Protein-to-Energy Ratios in Artificial Diets

**DOI:** 10.3390/ijms20235983

**Published:** 2019-11-28

**Authors:** Muhammad Shoaib Alam, Xu-Fang Liang, Liwei Liu, Shan He, Yulan Kuang, Seyed Hossein Hoseinifar, Farman Ullah Dawar

**Affiliations:** 1 College of Fisheries, Chinese Perch Research Center, Huazhong Agricultural University, Wuhan 430070, China; Shoaibalam530@gmail.com (M.S.A.); heshan@mail.hzau.edu.cn (S.H.); kyl1551789433@163.com (Y.K.); 2 Innovation Base for Chinese Perch Breeding, Key Lab of Freshwater Animal Breeding, Ministry of Agriculture, Wuhan 430070, China; 3 Department of Fisheries, Faculty of Fisheries and Environmental Sciences, Gorgan University of Agricultural Sciences and Natural Resources, Gorgan, 4918943464, Iran; hossein.hoseinifar@gmail.com; 4 Department of Zoology, Kohat University of Science and Technology (KUST), Kohat, Khyber Pakhtunkhwa 26000, Pakistan; farmandawar@kust.edu.pk

**Keywords:** Chinese perch, protein/energy ratio, body composition, growth and metabolism, protein synthesis, energy homeostasis, AMPK, mTOR pathways, nitrogen metabolism

## Abstract

The effect of dietary nutrients on novel farm species has always garnered wide research and economic interest. Chinese perch, an economically important carnivorous fish, accepts an artificial diet after taming, so it is essential to evaluate and optimize the nutritional and metabolic demands of this species. However, little is known about the effect of an artificial diet on the growth and metabolism of Chinese perch. Therefore, the present study evaluated the growth and metabolic responses of Chinese perch to experimental diets with different dietary protein/energy (P/E) ratios. Five isoenergetic diets (18 kJ/g) with graded levels of P/E ratios of 30.58, 33.22, 35.90, 38.6, and 41.35 mg/kJ (named A, B, C, D, and E) were formulated. A total of 225 Chinese perch (64.89 ± 0.28 g) were divided into five groups (triplicate tanks for each group), distributed into 15 (350 L) fiberglass tanks, and fed twice a day at 4% of fish wet body weight with the respective P/E ratio diets for 10 weeks. Compared with the other groups, Chinese perch in Group C showed significantly improved growth performance, weight gain (WG), specific growth rate (SGR), viscerosomatic index (VSI), hepatosomatic index (HSI), intraperitoneal fat (IPF), feed utilization, feed intake (FI), feed conversion ratio (FCR), protein efficiency ratio (PER), protein retention efficiency (PRE), energy retention efficiency (ERE), and feed efficiency (FE) as well as whole-body, muscle, and liver composition. Chinese perch in Group A, on the other hand, had the lowest growth performance, feed utilization, and body composition compared with the other groups. The activities of nitrogen metabolism-related enzymes (alanine aminotransferase (ALT), aspartate aminotransferase (AST) glutamate dehydrogenase (GDH), and adenosine 5′-monophosphate deaminase (AMPD)) as well as the mRNA expression of the *GDH* and *AMPD* genes were significantly lower than those in the other groups. Similarly, the expression of *NPY* and *AgRp* were significantly higher in Group C compared with the other groups. However, the gene expression of *CART* and *POMC* was not affected by the dietary P/E ratios. In Group A, the expression of *mTOR*, *S6K*, and *4EBP1* was significantly lower and that of *AMPK*, *LKB1*, and *eEF2* was significantly higher when compared with the other groups. Biochemical analysis of blood showed that ALT, AST, total protein (TP), alkaline phosphatase (ALP), glucose (GLU), blood urea nitrogen (BUN), and triglyceride (TG) levels were also affected by the dietary P/E ratio. From our results, we concluded that Chinese perch growth performance and nutrient metabolism were significantly affected by the P/E ratio of the artificial diet. Second-order polynomial regression analysis revealed that Chinese perch growth performance was optimal at a P/E ratio of 37.98 in the artificial diet.

## 1. Introduction

Chinese perch (*Siniperca chuatsi*) is a carnivorous freshwater fish that is widely cultured in China and Asia in general [1]. Chinese perch is commercially important because of its rapid growth, meat quality, and environmental adaptability [1]. Chinese perch normally feed on live fish [2] and have higher dietary protein requirements than other fish [3,4]. In aquaculture, feed cost is 60–70% of the total operational cost, and protein is an essential and expensive component [5]. Although dietary protein is significant for the growth and metabolism of carnivorous fish, excessive dietary protein can be metabolized as an energy source, resulting in increased feed cost and nitrogenous waste that potentially impairs fish growth and metabolism [6]. A balanced ratio of dietary protein to non-protein energy can spare dietary protein from energy metabolism and improve protein utilization and fish growth [7,8]. Dietary protein consumption may be improved by replacing some dietary protein with carbohydrates or lipids to take advantage of the protein-sparing effect [9]. However, an optimal balance between dietary protein and energy components is essential since the surplus or absence of non-protein energy results in the lower utilization of dietary protein and energy, potentially reducing fish growth [10,11,12].

A balanced dietary P/E ratio is necessary for the regulation of food intake and metabolism of nutrients including carbohydrates and lipids [13,14]. High feed intake improves fish growth and directly correlates with increased oxidative metabolism and protein synthesis as a result of surplus amino acids [15]. Surplus amino acids from a protein-rich diet cannot be directly stored in the fish body; they are deaminated and transformed into compounds used for energy [16,17]. In fish, intermediary metabolism and enzyme expression are modulated by nutritional status [18], and nitrogen metabolism is influenced by the dietary P/E ratio [19]. Alanine aminotransferase (ALT), aspartate aminotransferase (AST), and glutamate dehydrogenase (GDH) play important roles in nitrogen metabolism and the production of plasma ammonia, and their activities influence the levels of blood metabolites [20,21]. Imbalances in dietary nutrients also affect the specific enzyme activities required for deamination and transamination [22,23]. Adenosine 5′-monophosphate deaminase (AMPD) catalyzes the irreversible hydrolysis of adenosine monophosphate (AMP) to inosine monophosphate (IMP) and ammonia [24]. AMPD produces most ammonia in the white muscle of fish [25], and is an essential part of the purine nucleotide cycle [24]. Therefore, such enzyme activities have been used as indicators of nitrogen metabolism.

Furthermore, the dietary P/E ratio affects nutrient metabolism at the cellular level in the fish body. Cell growth depends on protein synthesis and requires energy, and protein synthesis and energy homeostasis are regulated by the mechanistic target of rapamycin (mTOR) and adenosine 5′-monophosphate (AMP)-activated protein kinase (AMPK) pathways, respectively [26,27]. The mTOR pathway contains two complexes, mTORC1 and mTORC2 [28], which regulate growth factors [29,30] and promote cell survival, respectively [31]. Two downstream signaling substrates of mTORC1—4EBP1 and S6 kinase—jointly regulate mRNA transcription and translation. During protein synthesis, activated mTORC1 phosphorylates and activates S6K and 4EBP1. Then, S6K promotes ribosomal protein expression and translation-regulating proteins to regulate protein synthesis, and nonphosphorylated 4EBP1 binds to eIF4E to inhibit protein synthesis. When 4EBP1 is phosphorylated by active mTOR, it dissociates from eIF4E, which then binds to other translation initiation factors to initiate protein synthesis [32]. Similarly, AMPK is activated and phosphorylated by the upstream kinase LKB1 during energy stress conditions [33]. The activation agents of AMPK induce the phosphorylation of eEF2, which then decreases protein synthesis at the elongation stage. Thus, the AMPK and mTOR pathways are two critical regulators of protein synthesis and energy homeostasis; in mammals, their inter-relationship is well-established [34,35,36].

Given the effect on growth and protein retention, studies have addressed the importance of the dietary P/E ratio in various fish species such as sea bream (*Sparus aurata*) [37], rainbow trout (*Oncorhynchus mykiss*) [38], parrotfish (*Oplegnathus fasciatus)* [39], red-spotted grouper (*Epinephelus akaara)* [40], and Nile tilapia (*Oreochromis niloticus*) [41]. However, the dietary P/E ratio varies with many factors such as species, fish size, fish weight, nutrient digestibility, dietary protein sources, protein requirements, life stages, feeding ratio, experimental design, and environmental conditions. Additionally, no studies have evaluated the P/E ratio of the artificial diet of Chinese perch, and most studies on the P/E ratio diet have only focused on growth performance and feed utilization. Limited studies have investigated the further effect of P/E ratio diets on nitrogen metabolism, protein synthesis, and energy homeostasis in the fish body at the molecular level. Therefore, in this context, this study aimed to evaluate the optimal P/E ratio and the growth and metabolic response of Chinese perch to different dietary protein-to-energy ratios in artificial diets.

## 2. Results

### 2.1. Growth Performance and Feed Utilization

**Figure 1:**
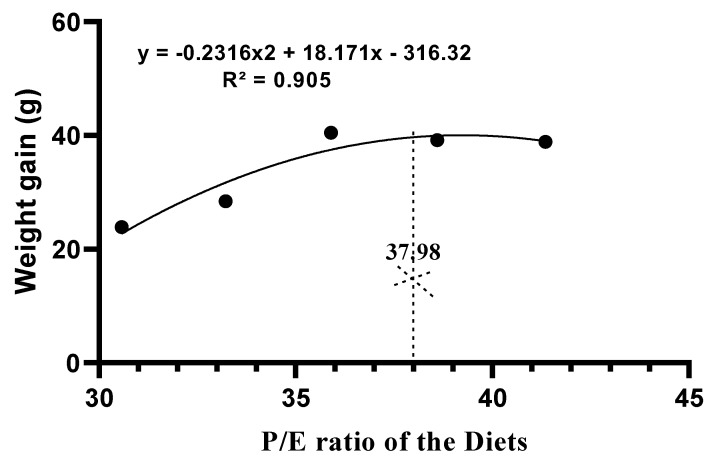
Second-order polynomial-regression analysis of the weight gain of Chinese perch fed with diets with different P/E ratios.

Growth performance and feed utilization of Chinese perch fed with the experimental diets with different P/E ratios are shown in Table 1. Chinese perch in Group C showed the best growth performance and feed utilization in terms of weight gain (WG), specific growth rate (SGR), protein efficiency ratio (PER), feed efficiency, protein retention efficiency (PRE), and energy retention efficiency (ERE) when compared with the other groups. The feed conversion ratio (FCR) was significantly lower in Group C when compared with the other groups, while feed intake (FI) in Groups C and B was significantly lower when compared with that of the other groups. Similarly, the VSI and IPF were significantly lower in Group G when compared with those in the other groups. However, the HSI in Groups A and E was significantly higher than that in other groups. A second-order polynomial regression of weight gain of the Chinese perch revealed that the best P/E ratio was 37.98 mg/kJ in this experiment (Figure 1).

### 2.2. Proximate Composition of Whole-Body, Muscle, and Liver Tissue

The proximate compositions of whole-body tissue and those of muscle and liver tissue are given in Table 2 and Table 3, respectively. Whole-body moisture, crude protein, lipids, and ash content were significantly affected by the P/E ratio of the experimental diets. Moisture and lipid contents of the Chinese perch were significantly higher in Group A, and protein and ash contents were significantly lower in Group A when compared with the other groups. Similarly, in muscle and liver, both moisture and lipid contents were significantly higher in Group A when compared with the other groups, and the trend observed for whole-body protein was similar to that for protein content in the muscle and liver. On the other hand, Group C showed significantly higher whole-body, muscle, and liver protein contents and lower moisture and lipid contents when compared with all other groups.

### 2.3. Enzyme Activities of Nitrogen Metabolism

**Figure 2:**
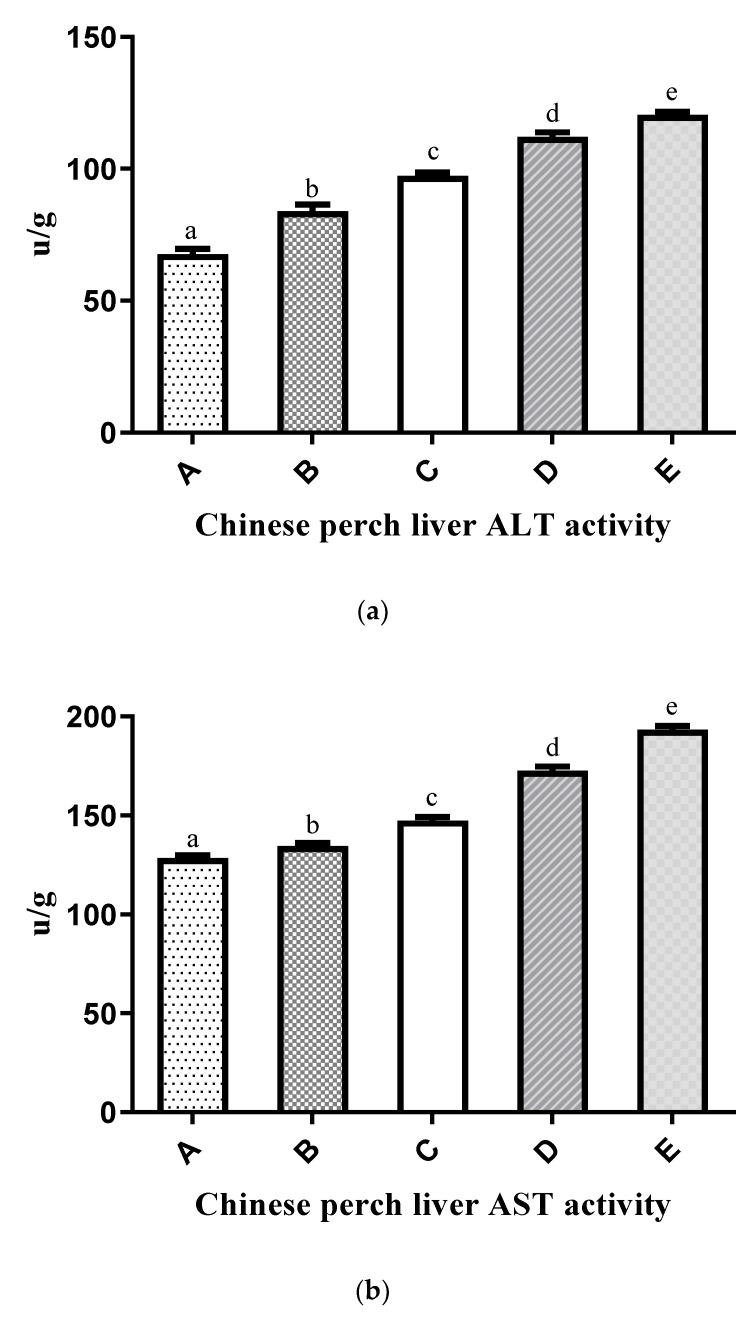

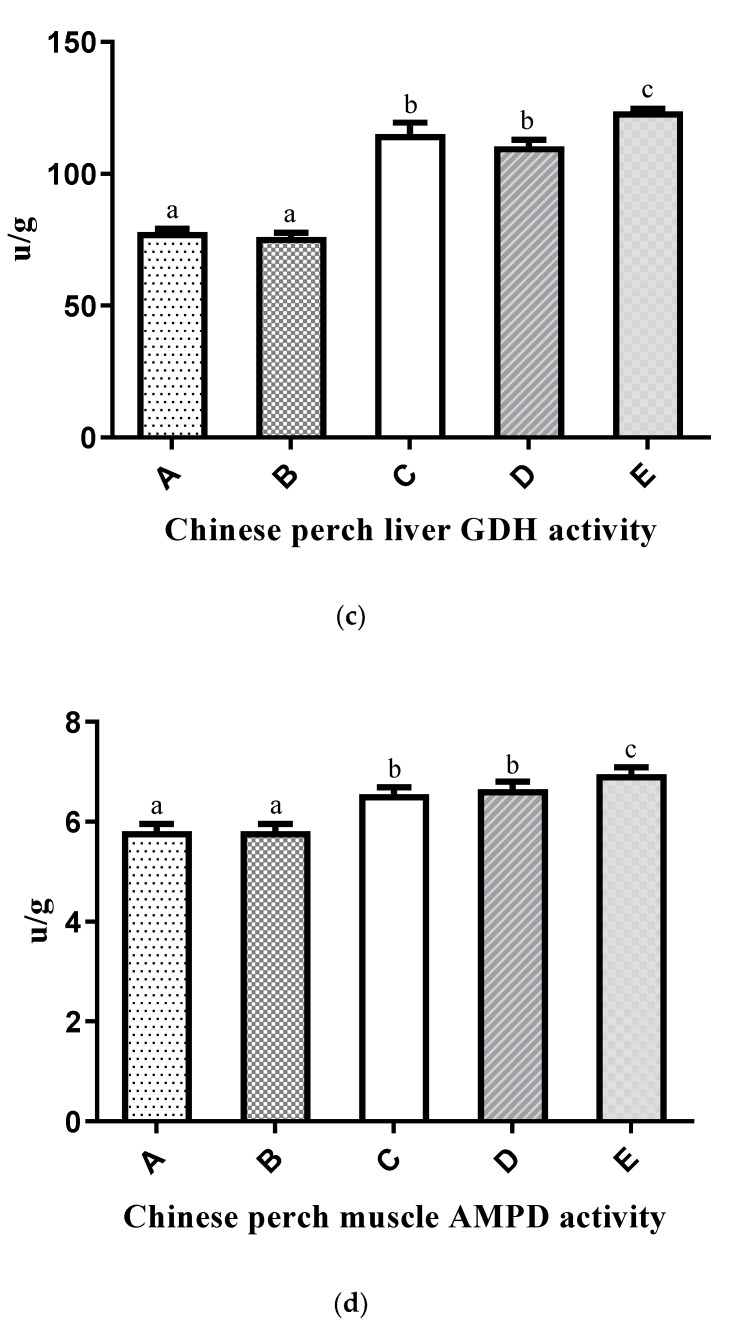
Effects of different P/E ratio diets enzyme activities involved in nitrogen metabolism in Chinese perch: (**a**) ALT, (**b**) AST, (**c**) GDH, and (**d**) AMPD. The x-axis shows different groups (A–E) of experimental diets, and the y-axis shows the enzyme activity level. Vertical bars with lowercase letters indicate a significant difference between treatments (*p* < 0.05).

The nitrogen metabolism enzyme activities of ALT, AST, and GDH in the liver and AMPD in muscle tissue are shown in Figure 2a–d. These enzymes were significantly affected by the P/E ratios in the experimental diets. Nitrogen metabolism enzyme activities in the liver (ALT, AST, and GDH) showed an increasing trend with increasing P/E ratios in the experimental diets. The activities of these enzymes in Group E were significantly higher than those in the other groups. Similarly, in muscle, AMPD activity had a significantly increasing trend with increasing P/E ratios in the experimental diets.

### 2.4. mRNA Expression of Nitrogen Metabolism Genes

**Figure 3:**
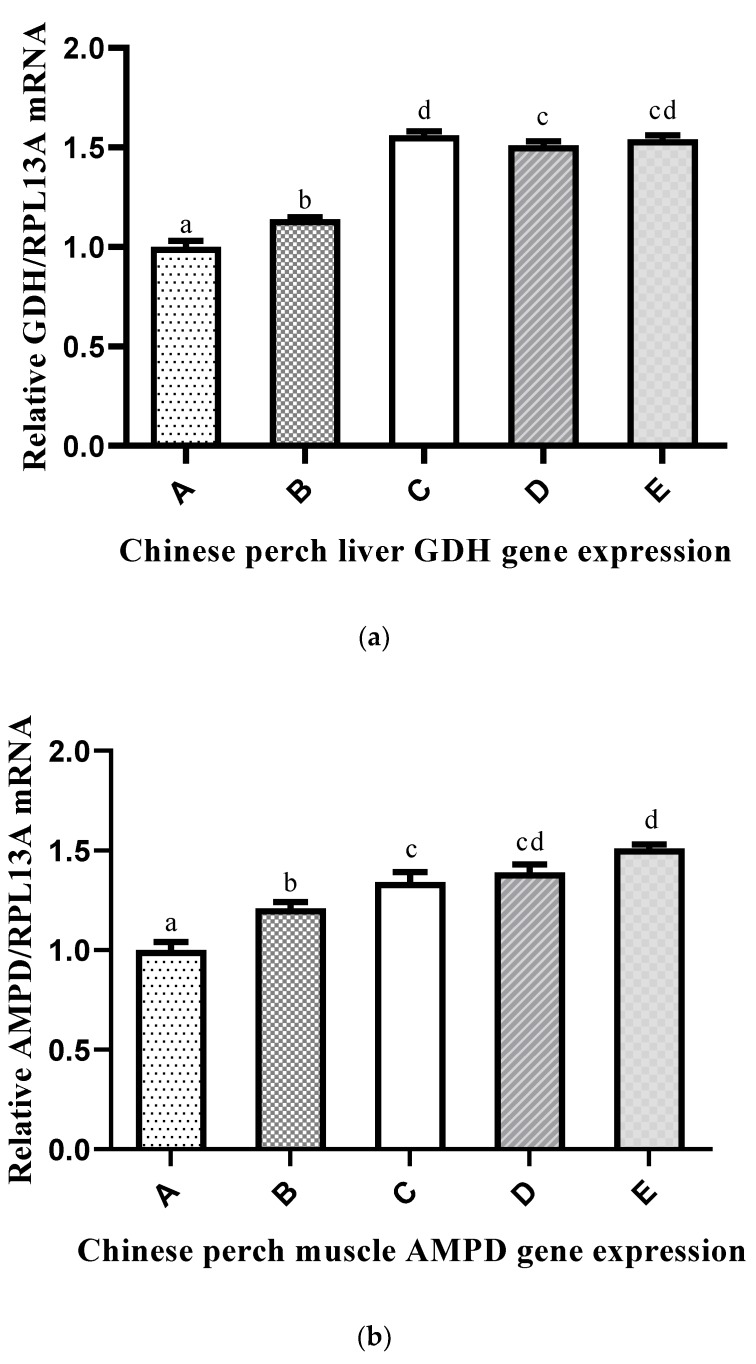
Effects of different P/E ratio diets on mRNA expression of nitrogen metabolism genes in Chinese perch: (**a**) *GDH* and (**b**) *AMPD*. The x-axis shows different groups (A–E) of experimental diets, and the y-axis shows the gene expression level. Vertical bars with lowercase letters indicate a significant difference between treatments (*p* < 0.05).

The mRNA expression levels of nitrogen metabolism genes (*GDH* and *AMPD*) in the liver and muscle of Chinese perch are shown in Figure 3a,b. The *GDH* expression level was significantly higher in Group C and lower in Group A compared with those of the other groups. The *AMPD* expression level in the muscle was significantly lower in Group A and significantly higher in Group E when compared with those in the other groups.

### 2.5. mRNA Expression of Appetite Regulation Genes

**Figure 4:**
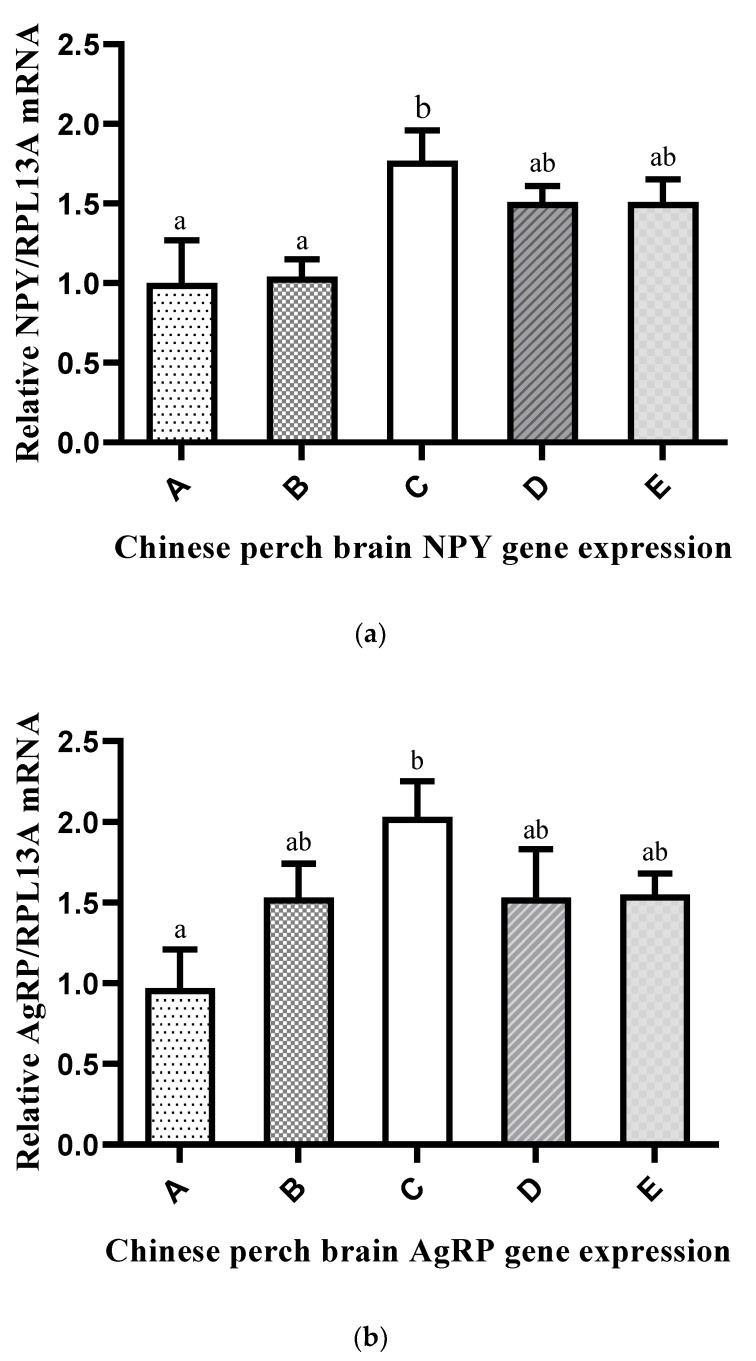

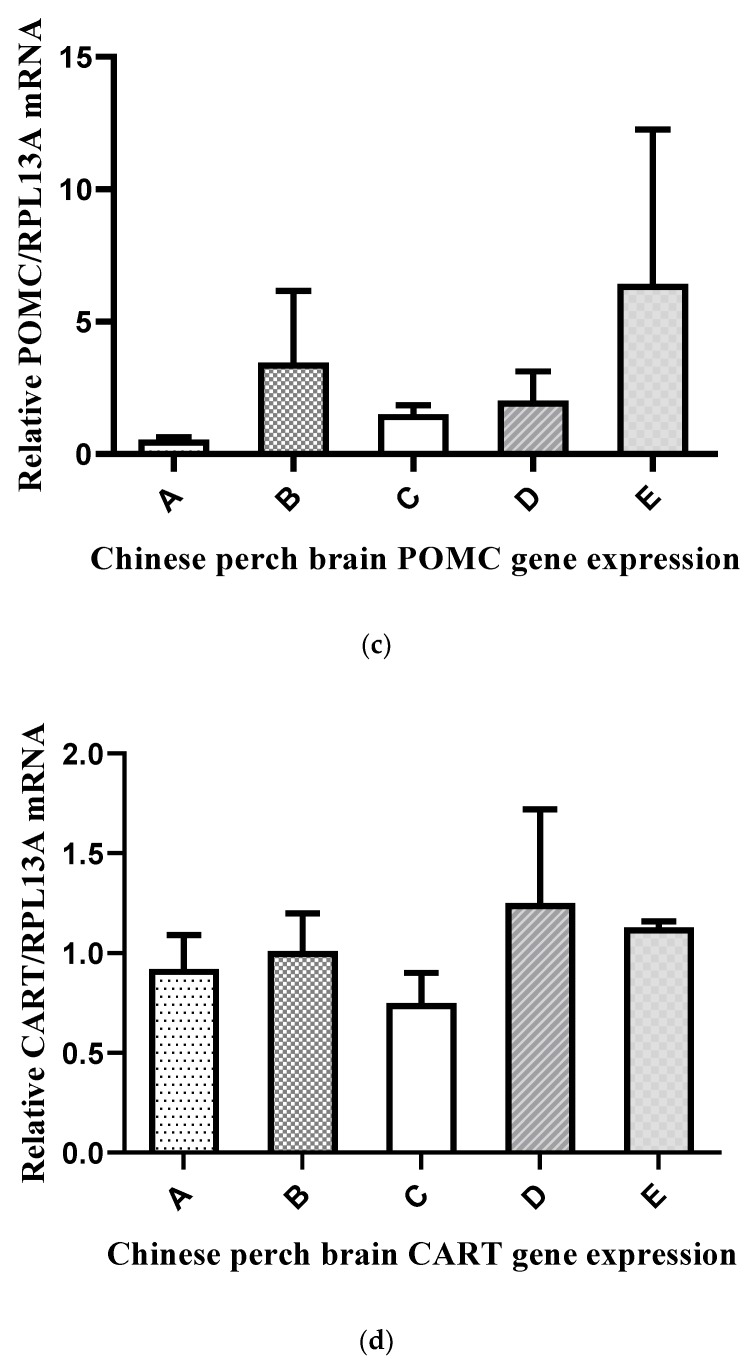
Effects of different P/E ratio diets on the mRNA expression of appetite regulation genes in Chinese perch: (**a**) *NPY*, (**b**) *AgRP*, (**c**) *POMC*, and (**d**) *CART*. The x-axis shows different groups (A–E) of experimental diets, and the y-axis shows the gene expression level. Vertical bars with lowercase letters represent a significant difference between treatments (*p* < 0.05).

The mRNA expression levels of appetite regulation genes (*NPY*, *AgRp*, *POMC*, and *CART*) in the brain of Chinese perch are shown in Figure 4a–d. The expression of the *NPY* and *AgRp* genes was significantly affected by the P/E ratio of the experimental diets. Compared with the expression levels in the other groups, the expression of both genes was significantly higher in Group C and significantly lower in Group A. On the other hand, the gene expression of *POMC* and *CART* were normally expressed and not affected by the P/E ratio of the experimental diets.

### 2.6. mRNA Expression of AMPK and mTOR Pathway Genes

**Figure 5:**
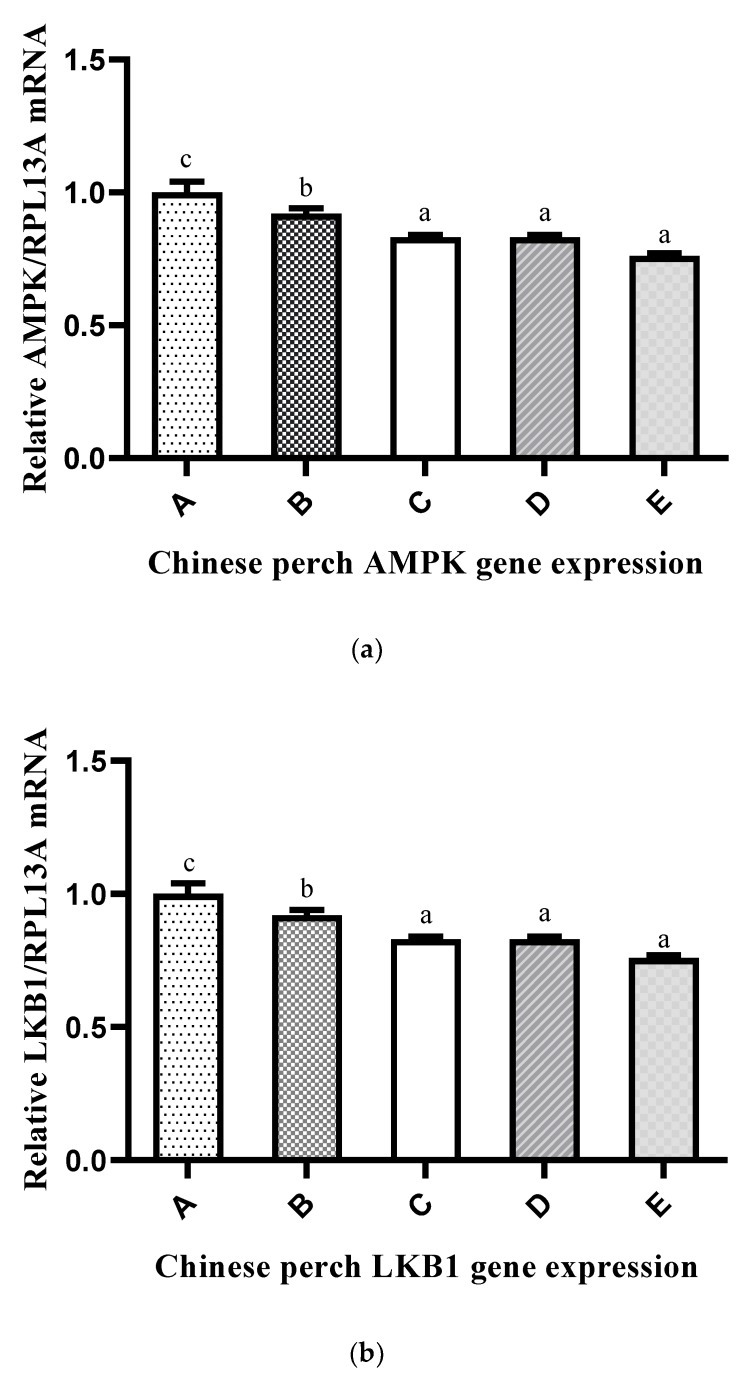

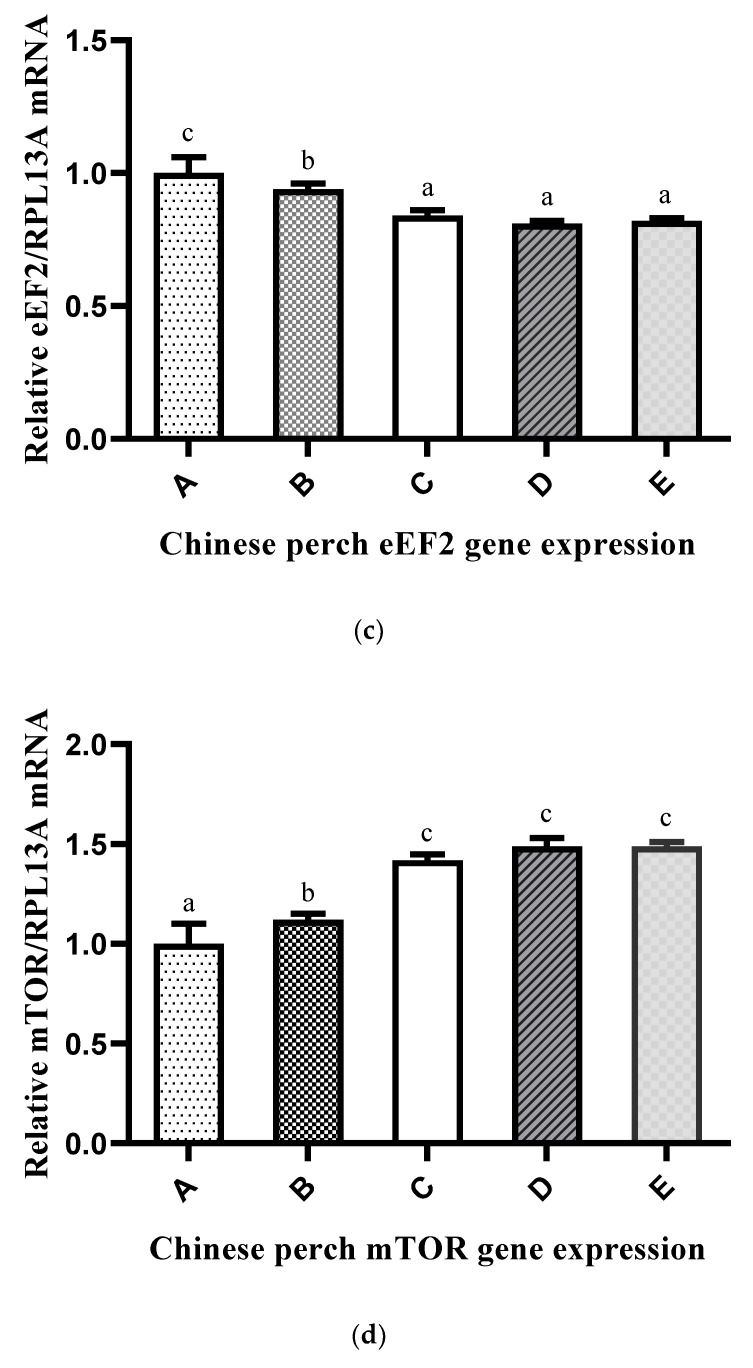

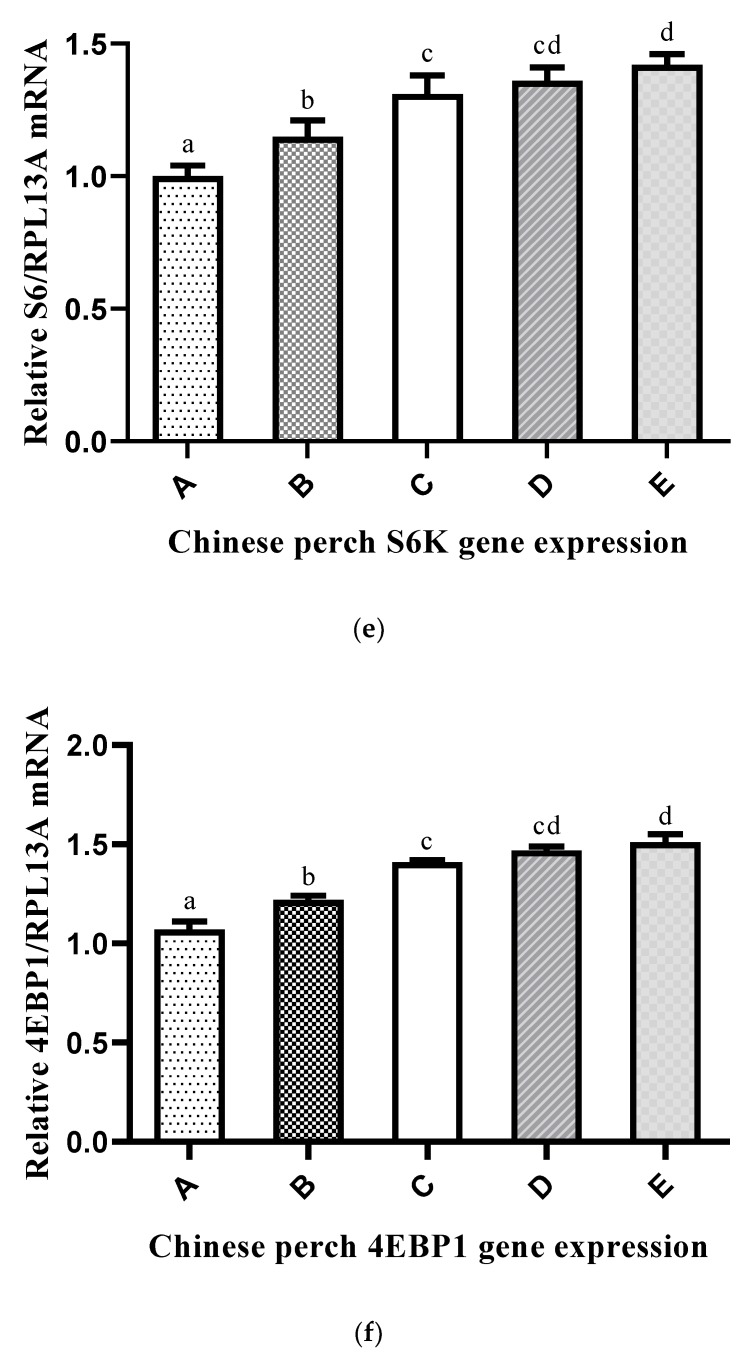
Effects of different P/E ratio diets on the mRNA expression of AMPK and mTOR pathway genes in Chinese perch: (**a**) *AMPK*, (**b**) *LKB1*, (**c**) *eEF2*, (**d**) *mTOR*, (**e**) *S6K*, and (**f**) *4EBP1*. The x-axis shows different groups of experimental diets, and the y-axis shows the gene expression level. Vertical bars with lowercase letters represent a significant difference between treatments (*p* < 0.05).

The mRNA expression levels of genes (*AMPK*, *LKB1*, *eEf2*) involved in the AMPK pathway and genes (*mTOR*, *S6K*, *4EBP1*) involved in the mTOR pathway are shown in Figure 5a–f, respectively. The expression of *AMPK*, *LKB1*, and *eEf2* was significantly higher in Group A when compared with that in the other groups, and there was no significant difference between Groups C, D, and E. However, the expression of *mTOR*, *S6K*, and *4EBP1* was significantly lower in Group A and higher in Group E when compared with that of the other groups.

### 2.7. Blood Biochemistry

Serum biochemical parameters were significantly affected by the experimental diets (Table 4). The GLU level was significantly lower in Group A compared to that in the other groups. The TP and TG levels showed an increasing trend in blood serum with the increasing P/E ratio in the experimental diets. ALT and AST were significantly higher in Groups A and E when compared with those in the other groups; however, the ALP level showed no difference between any of the groups.

## 3. Discussion

Fish food should be balanced in terms of proteins, carbohydrates, lipids, and other nutrients in order to sustain their stable growth [42]. The knowledge of these dietary nutrients is essential for the formulation of fish feed. Dietary protein, the most important and expensive part of fish feed, could be spared from energy metabolism by replacing it with non-protein energy sources to enhance fish growth and reduce feed cost [43]. In this study, the growth performance and metabolism of Chinese perch were significantly affected by the dietary P/E ratio of the experimental diets. The WG and SGR of the Chinese perch showed an increasing trend as the dietary P/E ratio increased to 35.90; after that, a slightly decreasing trend was observed as the dietary P/E ratio further increased. Generally, fish growth improves as the dietary protein increases to an optimal level, but dietary protein in excess of this level may have negative effects on fish growth and metabolism [44]. Accordingly, in the present study, the best growth performance and feed utilization were observed in Group C when compared with those in the other groups. In Group A (low P/E ratio), poor growth performance and feed utilization indicated lower protein availability for maintaining proper growth and metabolism [45]. Correspondingly, fish in Group E (high P/E ratio) had a lower weight gain when compared with Group C, which illustrates the catabolism of excess protein to metabolize excessive amino acids [46,47]. This may result in further energy costs through deamination and amino acid excretion [48,49,50]. This hypothesis is further supported by the lower PER, PRE, and ERE values observed in the present study in Group E (higher P/E ratio) when compared with those in Group A (lower P/E ratio diet). Fish cannot utilize excess dietary protein for protein synthesis, so most of that protein is utilized as an energy source in metabolism [51]. A similar trend was observed in bagrid catfish (*Pseudobagrus fulvidraco*) [52], *Spinibarbus hollandi* [49], silver barb (*Puntius gonionotus*) [53], two-banded seabream (*Diplodus vulgaris*) [54], tiger puffer (*Takifugu rubripes*) [55], and grass carp (*Ctenopharyngodon Idella*) [56].

Studies have established that dietary nutrients significantly influence fish health and nutrient utilization [57]. The VSI, HSI, and IPF ratio are good indicators of dietary nutrient utilization [58,59]. In the present study, the highest VSI, HSI, and IPF ratio were found in Groups A and E. This shows that the Chinese perch in these groups stored lipids from their diets in the abdomen and viscera and mainly used protein for energy purposes. Lipids and carbohydrates are excellent sources of non-protein energy for growth and metabolism, but their inclusion with protein at non-optimal proportions can affect feed consumption and body fat [60]. Similar to our findings, the results reported by Deng et al. (2011) showed that high HSI was associated with the protein/carbohydrate level or unsuitable protein/energy ratio [3] in *Polydactylus sexfilis*. Studies have also reported that fish that were fed diets low in protein experienced glycogen deposition in the liver, resulting in an increased HSI [61,62,63]. Typically, the ability of carnivorous fish to utilize dietary carbohydrates as an energy source is limited when compared with that of other fish, and their dietary carbohydrate/lipid ratio is often negatively related to their IPF ratio [63,64].

Feed intake was significantly higher in groups that were fed high P/E ratio diets compared with those fed low P/E ratio diets. Usually, feed intake regulation in fish depends on their metabolizable energy demands [54,65,66]. Similarly, if a diet is deficient in an essential nutrient, the fish tries to consume more feed to fulfill the demands for the specific nutrient [67]. Sá et al. (2014) [67] reported that low-protein diets were poorly digestible. The hypothalamus controls food intake either by appetite regulation or by receptors at both the transcriptional and translational level [68,69,70]. The hypothalamus contains two major kinds of neurons that regulate and integrate metabolic and endocrine signals related to food intake and energy homeostasis: one is the appetite-stimulating (orexigenic) neuron that co-expresses neuropeptide Y (NPY) and Agouti-related peptide (AgRP) [71], and the other is the appetite-suppressing (anorexigenic) neuron that co-expresses pro-opiomelanocortin (POMC) and cocaine- and amphetamine-regulated transcript (CART) [72]. In the present study, the mRNA expression of the *NPY* and *AgRp* genes was significantly affected by the dietary P/E ratio of the experimental diets. The gene expression level linearly increased with the increasing P/E ratio in the diets. The highest gene expression was found in Group C, followed by Groups D and E. However, the expression of the *POMC* and *CART* genes was not significantly affected by the P/E ratio in the experimental diets. This indicates that Chinese perch use high dietary protein for energy and body homeostasis regulation. Similarly, in Groups A and B, the protein level was below the optimal level, which could be the reason for the low feed intake and acceptability; that is, the feed was low in dietary protein and high in carbohydrates/lipids.

The protein/energy ratio can affect the growth performance, feed efficiency, and body composition of fish. Fish body composition could be affected by protein synthesis, protein deposition rate, and protein related transcriptome changes [73,74,75]. In the present study, the moisture content in the whole body, muscle, and liver were negatively related to dietary P/E ratios. The protein and ash content in the whole body, muscle, and liver increased from Groups A to C; after that, a slight decrease was observed from Groups D to E. However, the lipid contents of the whole body, muscle, and liver were significantly higher in Groups A (low P/E ratio) and E (high P/E ratio). Moisture contents and lipids have a direct relationship, and the increasing dietary P/E ratio positively affected lipid deposition and negatively affected moisture content [76,77]. Similarly, in their study on the orange-spotted grouper (*Epinephelus coioides*), Luo et al. (2004) [78] reported that crude whole-body and muscle protein was positively correlated with dietary protein content. Similar results were also reported in mahseer (*Tor putitora Hamilton*) [79], olive flounder (*Paralichthys olivaceus*) [30], *Zacco barbata* [80], silver perch (*Bidyanus bidyanu*) [61], and in black sea bream (*Sparus macrocephalus*) [81]. In contrast, body protein content linearly decreased in *E. malabaricus* when dietary protein increased [82]. Furthermore, Lee et al. (2003) [83] and Schulz et al. (2007) [84] found no significant body protein changes with dietary protein-level fluctuations. Body protein deposition improves with the appropriate dietary protein to some extent (optimal level). After that level, increasing the dietary protein affects body protein deposition, growth performance, and metabolism, as observed in the fish that were fed a high P/E ratio diet (Group E). Moreover, in the present study, lipid contents in the whole body, muscle, and liver were significantly higher in the low and high P/E ratio diet groups (A and E) when compared with those of the other groups. This indicates that at a low P/E ratio diet, the protein was below the optimal level and mostly used for body protein requirements, and dietary lipids and carbohydrates were not efficiently used for energy purposes. However, in higher P/E ratio diet groups, excess dietary energy was stored as lipids in the body. Similarly, the increase in dietary protein level was associated with increased body lipid content; similar results were also reported by Shiau et al. (1996) [85], Bai et al. (1999) [86], and Kim et al. (2004) [87]. However, conflicting results were reported by Chen et al. (1994) [88] and Kim et al. (2002) [30]. In a study on red drum (*Sciaenops ocellatus*) by McGoogan and Gatlin (1999) [89], neither dietary protein nor energy levels significantly affected the lipid content in muscle.

The nutritional status in fish can be modulated by the expression of key enzymes involved in intermediary nitrogen metabolism [20,21]. Transaminase and deaminase enzyme activities have been useful in evaluating the feeding status of fish [21,90,91]. Amino acid-metabolizing enzyme levels and nitrogen excretion are reliable indicators of dietary protein utilization, and amino acid metabolism is involved in deamination and transamination reactions. In the present study, the P/E ratio in the experimental diets significantly affected ALT, AST, and GDH in the liver and AMPD in the muscle. These enzymes (ALT, AST, GDH, and AMPD) and the mRNA expression of *GDH* and *AMPD* showed a significant increasing trend with the increasing dietary P/E ratio. The upsurge of ALT, AST, and GDH activities that were observed in Chinese perch liver may reflect the use of excess hydrocarbons from amino acids as sources to meet energetic demands. Similar results have been reported when dietary protein/carbohydrate ratios were increased. ALT and AST activities were increased in the liver of *Sparus aurata* [20], and similar results were observed for ALT in *O. mykiss* [91], in AST and ALT in *R. quelen* [21], and in the GDH activity of the European eel (*Anguilla Anguilla*) [92]. The increase in hepatic activity of protein-metabolizing enzymes associated with high P/E ratio diets may indicate the use of excess protein as an energetic compound. *GDH* gene expression was also high in groups fed high P/E ratio diets. Correspondingly, increasing gene expression levels with increasing protein levels were also observed by Liu et al. (2012) [93] in triploid fish. The skeletal muscle of fish produces ammonia by deamination, decomposition of glutamine, and trans-deamination, all of which contribute to the purine nucleotide cycle [94]. For example, in the purine nucleotide cycle, AMPD catalyzes the irreversible hydrolysis of AMP to IMP and ammonia. In our study, the enzyme activity and gene expression of AMPD gradually increased with the increase in dietary P/E ratio. The increase in AMPD activity may contribute more ammonia to the muscle through the purine nucleotide cycle. Muscle AMPD activity in fish fed with high P/E ratio diets was significantly higher than that in fish fed with low P/E ratio diets. Therefore, when dietary P/E ratios were higher than optimal, ammonia production and muscle quality were affected.

TOR is a key promoter of cell growth in response to growth factors and nutrients, while AMPK is a critical sensor and regulator that responds to homeostatic cell energy signals. Under high-nutrient and high-energy conditions, TOR promotes anabolism, whereas AMPK promotes catabolism in low-nutrient and low-energy conditions [33]. In the present study, the expression of the *mTOR*, *S6K*, and *4EBP1* genes, whose protein products are involved in TOR pathways, was significantly lower in Group A when compared with the other groups. *AMPK*, *LKB1*, and *eEEf2*, on the other hand, are involved in AMPK pathways, and their expression was significantly higher in Group A compared with the other groups. The TOR pathway regulates protein synthesis through its downstream targets S6K and 4EBP1 [95], whereas the activation of AMPK relies on the decrease in the cellular ATP/GTP level and the increase in the cellular AMP level during hypoxia or nutrient deprivation conditions. Studies have reported that LKB1 is a major kinase that is responsible for the phosphorylation and activation of AMPK during energy stress conditions [96]. Additionally, AMPK stimulates eEF2K activity during protein synthesis, which is a physiologically logical response to the slowdown of protein synthesis during energy or nutrient deficiency [27]. The AMPK activation of eEF2K leads to the deactivation of the phosphorylation of eukaryotic elongation factor 2 (eEF2) and the inhibition of the TORC1 effector and protein synthesis [36,97]. These expression patterns of genes in the mTOR–AMPK pathway show the clear relationship between pathway activation and inhibition, and nutrient levels. When the fish were fed with low P/E ratios diet, the expression of genes in the mTOR pathway was lower, and the expression of genes in the AMPK pathway was higher, indicating that low protein synthesis resulted in low growth performance and poor metabolism; the opposite trend was observed when fish were fed with high P/E ratio diets.

Studies have reported that feeding treatments, stress, and disease can alter blood characteristics in fish [98]. In the present study, dietary P/E ratio affected the biochemistry of blood serum in Chinese perch. Blood ALT and AST were significantly higher in Groups A and E when compared with those in the three other groups. This may be an indication of liver injuries or dysfunction since blood AST and ALT levels reflect these conditions [99]. Blood TP and TG were increased with the increase in the P/E ratio of the experimental diets. These results agree with those reported by Yengkokpam et al. (2016) for the blood TP in Rohu (*Labeo rohita*) [100] as well as those found for the blood TG in Nile tilapia (*Oreochromis niloticus*) [73] and European eel [92]. In accordance with studies in *Dicentrarchus labrax* [101] and *Rhamdia quelen* [21], blood BUN levels were positively increased with an increased dietary P/E ratio in the experimental diets. These increases may be due to the conversion of amino acids into carbohydrates or a smaller amount of fats [102]. Correspondingly, the blood GLU level also positively increased with an increase in dietary P/E ratio in the experimental diets. The rise in the GLU level suggests that gluconeogenesis may occur after the ingestion of increased dietary protein levels [74,79].

## 4. Materials and Methods

### 4.1. Ethical Approval

All experimental procedures followed the Ethical Guidelines of “Institutional Animal Care and Institute of Huazhong Agricultural University” (Ethical Approval No. HBAC20091138; date, 15 November 2009; Wuhan, China).

### 4.2. Experimental Diets

Table 5 details the compositions of the experimental diets used in this study. Five isoenergetic diets (18 kJ/g) were formulated with varied P/E ratios (30.58, 33.22, 35.90, 38.60, and 41.35 mg/kJ) through graded protein levels (38.66%, 41.90%, 44.78%, 48.04%, and 51.71%) and named A, B, C, D, and E. The dietary ingredients were procured from Gaolong Feed Technology Co. Ltd. (Wuhan, China). First, dietary ingredients were ground into powder, passed through a 300 µm mesh, mixed well, and then pelleted into 3 mm feed pellets by a laboratory pelleting machine. After that, the pellets were air-dried at room temperature, transferred into plastic bags, and stored in a deep freezer at −20 °C for further use.

### 4.3. Chinese Perch Culture and Feeding Trial

Chinese perch were transported from an agricultural development company in Wuhu. Prior to the experiment, all fish were acclimatized to the experimental conditions and weaned onto the artificial diets for four weeks [2]. After acclimatization, a total of 225 fish were randomly selected and distributed into five groups of 15 tanks (350 L). Each group was assigned to triplicate tanks, and each tank was stocked with 15 fish (fish weight: 64.89 ± 0.28 g) fed with the respective P/E ratio diets. During the culture period, fish were fed twice a day at 08:00 and 17:00 at 4% of fish wet body weight. Throughout the culture period, the filtered water flow rate was 3 L min^−1^, the photoperiod was 12/12 h (light/dark), the pH value was around 7.81 ± 0.21, the ammonia content was approximately 0.18 ± 0.04 mg/L, the dissolved oxygen (DO) was about 7 mg/L, and the water temperature was around 24 ± 2 °C.

### 4.4. Sample Collection

Prior to the experimental trial, a total of nine fish were selected for whole-body and tissue analysis for the calculation of protein retention efficiency and energy retention efficiency. At the end of the 10-week feeding trial, Chinese perch were starved for one day and euthanized with 75 mg/L tricaine methane-sulfonate (Argent Chemical Laboratories, Redmond, WA, USA). Then, the fish were counted and weighed for growth performance. Afterward, nine fish/group (three fish/tank) were chosen for whole-body chemical analysis and stored at −20 °C. Nine more fish/group (three fish/tank) were chosen for the analysis of blood, muscle, and liver tissue samples (enzyme and chemical analysis) and measurement of viscerosomatic (VSI), hepatosomatic (HSI), and intraperitoneal fat (IPF) indices. Blood samples were taken from the caudal vein of the fish and stored at 4 °C overnight and then centrifuged at 2500× *g* for 20 min. The separated serum was sampled and stored at −20 °C. Muscle and liver tissue samples were taken by dissection, and the viscera were separated for VSI, HSI, and IPF measurement. Nine additional fish/group (three fish/tank) were chosen for the collection of brain, liver, and muscle tissue samples for gene expression analysis. These samples were immediately frozen in liquid nitrogen and stored at −80 °C for further gene expression analysis.

### 4.5. Proximate and Chemical Analyses

Proximate composition analyses of the experimental diets, fish body, and muscle and liver tissue were performed according to standard procedures (AOAC, USA, 1995) [103]. In brief, moisture content was assessed by oven-drying at 105 °C for 24 h. Ash content was assessed by muffle furnace incineration at 550 °C for 12 h. Crude protein content (N × 6.25) was assessed by the Kjeldahl procedure (Kjeltec 2300 Analyzer, Foss Tecator, Höganäs, Sweden). A Soxtec System HT (Soxtec System HT6, Tecator, Sweden) was used for lipid quantification by the ether extraction method. Gross energy content was assessed by using a 6200 Isoperibol Calorimeter (Parr Instrument Company, Moline, IL, USA) for the bomb calorimetry procedure.

### 4.6. Enzyme Activity Assay

The procedure of Valente et al. (2007) [104] was followed to obtain homogenates of liver and muscle samples. The Bradford (1976) [105] method was used to quantify the soluble protein content in the homogenates using bovine serum albumin (BSA) as a standard.

The activities of nitrogen metabolism enzymes (ALT, AST, GDH, and AMPD) were examined on the supernatant by a spectrophotometric technique. ALT (EC 2.6.1.2) and AST (EC 2.6.1.1) activities were examined by a commercial kit (Nanjing Jiancheng Biochemical Reagent Co., Nanjing, China) following the manufacturer’s instructions. GDH (EC 1.4.1.3) activity was measured by using a modified version of the Bergmeyer (1978) procedure [106], according to the instructions of the GLDH ELISA kit (Lai-Er Bio-Tech Company, Hefei, China). The AMPD enzyme was measured with a fish AMPD ELISA kit (Lai-Er Bio-Tech Company, Hefei, China) following the manufacturer’s protocol. Enzyme activity units are presented as U/g.

### 4.7. RNA Isolation and Reverse Transcription

Liver and muscle tissue RNAs were extracted by TRIzol reagent (TaKaRa, Tokyo, Japan) following the manufacturer’s protocol. RNA purity and integrity were assessed by gel electrophoresis, and the concentration of RNA samples was determined on a BioTek Synergy™ 2 Multimode 200 Microplate Reader (BioTek Instruments, Winooski, VT, USA). Afterward, cDNA was obtained by using reverse transcriptase SuperScript™ II RT (Takara, Tokyo, Japan).

### 4.8. Real-Time qPCR Analysis

Real-time qPCR was used for the gene expression assay. All primers used in this study are shown in Table 6. All gene sequences of Chinese perch were obtained from our transcriptome-sequencing library [107]. Gene-expression assays were performed as described elsewhere [1], and the beta-actin gene (RPL13A) was used as an internal control. A quantitative thermal cycler (MyiQTM 2 Two-Color Real-Time PCR Detection System, Bio-Rad, USA) was used for gene expression assays. A 20 µL reaction was used that contained 10 µL of Green Master Mix AceQ^®^ qPCR SYBR^®^ (Vazyme, China), 8.2 µL of sterile double-distilled water, 1 µL of cDNA, and 10 mM each of forward and reverse primers of 0.4 µL. The PCR parameters were as follows: initial temperature of 95 °C for 5 min, followed by 40 cycles at 95 °C for 10 s, then 65 °C for 30 s, and a melt curve step (0.5 °C/second, gradually increasing to 95 °C with data acquisition every 6 s). Each sample was analyzed in triplicate. Analysis of gene expression with a housekeeping gene was performed by a comparative Ct (2^−ΔΔ*C*t^) value procedure [108]. All data are presented as the mean ± SEM.

### 4.9. Calculation and Formulas

The variables were calculated by the following equations:

Weight gain (%) = [(average final body weight (g) − average initial body weight (g))/average initial body weight (g)] × 100

Weight gain (g) = average final body weight (g) − average initial body weight (g)

Weight gain rate (g per fish) = average initial weight (g)/average fish weight gain (g)

Feed intake (g per fish per day) = total feed consumed (g, dry weight)/fish number/number of days

Specific growth rate (% day^−1^) = [(ln final body weight (g) − ln initial body weight (g))/number of days] × 100

Feed conversion ratio (g, dry-matter basis) = total feed consumed (g)/total weight gain (g)

Protein efficiency ratio = wet weight gain (g)/total fed protein (g)

Feed efficiency (%) = [total wet weight gain (g)/total feed consumed (g)] × 100

Protein retention efficiency (%) = [(final body protein (g) − initial body protein (g))/total fed protein (g)] × 100

Energy retention efficiency (%) = [Energy gain/Energy intake] × 100

Viscerosomatic index (VSI, %) = 100 × [viscera weight (g)/wet body weight (g)]

Hepatosomatic index (HIS, %) = 100 × [liver weight (g)/wet body weight (g)]

Intraperitoneal fat = 100 × [intestinal fat (g)/wet body weight (g)]

### 4.10. Statistical Analysis

SPSS (version 21) was used for all statistical analyses. Data are presented as mean ± SEM values. The Shapiro–Wilk and Levene tests were used to assess data normality and variance homogeneity. One-way analysis of variance (ANOVA) and Duncan’s multiple range tests were used for mean differences at the 5% level of statistical significance. Second-order polynomial regression analysis was used to determine the best P/E ratio of the experimental diet, which was considered to be the diet at which the maximum weight gain was obtained.

## 5. Conclusions

The results of this study demonstrate that the dietary P/E ratio in artificial diets significantly affects the growth and metabolism of Chinese perch. The optimal dietary P/E ratio for optimal growth was 37.98 mg/kJ. The dietary P/E ratio in artificial diets affects protein synthesis, energy homeostasis, enzyme activities, and serum biochemistry, which regulate growth performance, feed intake and utilization, and body composition in Chinese perch. An imbalanced dietary P/E ratio causes increased body fat and affects muscle quality as a result of excess nitrogen metabolism. The results of this study will help in the development of a balanced artificial diet for Chinese perch cost-effective aquaculture production.

## Figures and Tables

**Table 1:** Growth performance and feed utilization of Chinese perch fed with various P/E ratio diets.

Growth Index	A (P/E: 30.58)	B (P/E: 33.22)	C (P/E: 35.90)	D (P/E: 38.60)	E (P/E: 41.35)
Initial weight	64.65 ± 0.40	64.71 ± 0.34	64.48 ± 0.10	65.09 ± 0.11	65.52 ± 0.43
Final weight	108.80 ± 0.44 ^a^	115.80 ± 0.36 ^b^	130.76 ± 0.19 ^e^	127.02 ± 0.15 ^d^	124.31 ± 0.23 ^c^
Weight gain %	68.13 ± 0.6 ^a^	79.54 ± 0.35 ^b^	102.64 ± 1.37 ^e^	95.55 ± 0.80 ^d^	92.19 ± 0.08 ^c^
Weight gain	44.09 ± 0.37 ^a^	51.29 ± 0.24 ^b^	66.23 ± 0.53 ^e^	62.06 ± 0.07 ^d^	59.63 ± 0.14 ^c^
Weight gain rate	0.68 ± 0.01 ^a^	0.80 ± 0.02 ^b^	0.11 ± 0.01 ^e^	0.97 ± 0.01 ^d^	0.93 ± 0.02 ^c^
Feed intake	1.56 ± 0.12 ^b^	1.50 ± 0.05 ^a^	1.51 ± 0.18 ^a^	1.63 ± 0.01 ^c^	1.83 ± 0.06 ^d^
SGR (%/day)	0..74 ± 0.01 ^a^	0.84 ± 0.01 ^b^	1.01 ± 0.01 ^e^	0.96 ± 0.01 ^d^	0.93 ± 0.01 ^c^
FCR	2.48 ± 0.02 ^e^	2.06 ± 0.01 ^c^	1.59 ± 0.01 ^a^	1.84 ± 0.07 ^b^	2.16 ± 0.1**^d^**
PER	1.04 ± 0.01 ^a^	1.16 ± 0.01 ^b^	1.40 ± 0.1 ^e^	1.14 ± 0.01 ^d^	0.90 ± 0.02 ^c^
Feed efficiency	40.33 ± 0.28 ^a^	48.54 ± 0.31 ^c^	62.80 ± 0.53 ^e^	54.51 ± 0.03 ^d^	46.43 ± 0.15 ^b^
PRE	26.20 ± 0.21 ^b^	33.22 ± 0.38 ^d^	37.44 ± 0.07 ^e^	29.20 ± 0.13 ^c^	25.20 ± 0.08 ^a^
ERE	31.12 ± 0.10 ^b^	41.46 ± 0.43 ^c^	51.01 ± 0.05 ^e^	44.80 ± 0.03 ^d^	29.58 ± 0.10 ^a^
VSI	13.05 ± 0.32 ^c^	11.62 ± 0.37 ^ab^	10.91 ± 0.26 ^a^	11.18 ± 0.35 ^ab^	11.78 ± 0.10 ^b^
HSI	1.27 ± 0.04 ^b^	0.92 ± 0.03 ^a^	0.91 ± 0.03 ^a^	0.87 ± 0.01 ^a^	1.20 ± 0.01 ^b^
IPF	0.97 ± 0.11 ^b^	0.84 ± 0.04 ^ab^	0.74 ± 0.07 ^a^	0.80 ± 0.04 ^a^	0.88 ± 0.04 ^b^

Values are means ± SE; the different superscript letters ^a, b, c, d, e^ in the rows indicate a significant difference at *p* < 0.05.

**Table 2:** Whole-body proximate composition of Chinese perch fed with various P/E ratios.

	Whole Body			
Groups	Moisture	Protein	Lipids	Ash
**Initial**	79.30 ± 0.42	15.06 ± 0.07	3.13 ± 0.11	2.83 ± 0.08
**A**	75.03 ± 0.18 ^d^	16.46 ± 0.12 ^a^	4.52 ± 0.07 ^d^	3.59 ± 0.02 ^a^
**B**	74.53 ± 0.13 ^c^	17.36 ± 0.13 ^b^	4.07 ± 0.04 ^c^	3.83 ± 0.03 ^b^
**C**	73.47 ± 0.14 ^a^	18.52 ± 0.19 ^d^	3.30 ± 0.05 ^a^	4.63 ± 0.03 ^d^
**D**	74.07 ± 0.22 ^b^	18.09 ± 0.17 ^c^	3.62 ± 0.03 ^b^	4.14 ± 0.04 ^c^
**E**	74.37 ± 0.13b ^c^	17.59 ± 0.07 ^b^	4.15 ± 0.04 ^c^	3.82 ± 0.4 ^b^

Values are means ± SE; the different superscript letters ^a, b, c, d,^ in the rows represent a significant difference at *p* < 0.05.

**Table 3:** Muscle and liver proximate composition of Chinese perch fed various P/E ratios.

	Muscle			Liver		
Groups	Moisture	Protein	Lipids	Moisture	Protein	Lipids
**A**	78.07 ± 0.18 ^d^	17.45 ± 0.46 ^a^	1.7 ± 0.02 ^d^	83.72 ± 0.21 ^e^	10.99 ± 0.08 ^a^	2.6 ± 0.03 ^d^
**B**	76.53 ± 0.05 ^c^	18.19 ± 0.59 ^c^	1.51 ± 0.02 ^b^	82.03 ± 0.11 ^c^	11.59 ± 0.09 ^c^	2.19 ± 0.04 ^c^
**C**	75.16 ± 0.09 ^a^	18.83 ± 0.33 ^e^	1.22 ± 0.02 ^a^	80.24 ± 0.12 ^a^	12.46 ± 0.16 ^e^	1.86 ± 0.02 ^a^
**D**	75.54 ± 0.10 ^b^	18.51 ± 0.64 ^d^	1.59 ± 0.03 ^c^	81.03 ± 0.14 ^b^	11.98 ± 0.05 ^d^	2.06 ± 0.01 ^b^
**E**	76.28 ± 0.18 ^c^	17.97 ± 0.06 ^b^	1.62 ± 0.01 ^c^	82.42 ± 0.05 ^d^	11.30 ± 0.11 ^b^	2.24 ± 0.02 ^c^

Values are means ± SE; the different superscript letters ^a, b, c, d, e^ in the rows represent a significant difference at *p* < 0.05.

**Table 4:** Biochemical parameters of blood serum of Chinese perch fed with diets containing various P/E ratios.

Blood Indexes		A	B	C	D	E
1	ALT	13.00 ± 0.37 ^b^	9.67 ± 0.42 ^a^	10.50 ± 0.50 ^a^	10.33 ± 0.42 ^a^	13.17 ± 0.31 ^b^
2	AST	43.67 ± 1.09 ^b^	33.33 ± 0.42 ^a^	33.83 ± 0.70 ^a^	34.00 ± 0.86 ^a^	43.83 ± 1.38 ^b^
3	TP	37.30 ± 0.34 ^a^	41.33 ± 0.42 ^b^	43.83 ± 0.31 ^c^	44.87 ± 0.48 ^c^	46.67 ± 0.49 ^d^
4	ALP	50.67 ± 1.45	51.67 ± 2.19	50.33 ± 1.84	51.33 ± 1.78	50.83 ± 2.33
5	GLU	7.76 ± 0.08 ^a^	8.20 ± 0.15 ^b^	8.83 ± 0.19 ^c^	8.70 ± 0.07 ^c^	8.79 ± 0.19 ^c^
6	BUN	3.45 ± 0.12 ^a^	4.37 ± 0.23 ^b^	4.50 ± 0.06 ^b^	5.79 ± 0.34 ^c^	6.33 ± 0.17 ^c^
7	TG	1.46 ± 0.03 ^a^	1.69 ± 0.03 ^b^	1.78 ± 0.03 ^c^	1.91 ± 0.04 ^d^	1.96 ± 0.02 ^e^

Values are means ± SE; the different superscript letters ^a, b, c, d, e^ in the rows represent a significant difference at *p* < 0.05. Blood indexes: (1) alanine aminotransferase activity (U/L); (2) aspartate aminotransferase activity (U/L); (3) total protein (g/L); (4) alkaline phosphatase (U/L); (5) glucose (mmol/L); (6) blood urea nitrogen (mmol/L); (7) triglyceride (mmol/L).

**Table 5:** Chinese perch experimental diet ingredients and composition.

Ingredient	A (P/E: 30.58)	B (P/E: 33.22)	C (P/E: 35.90)	D (P/E: 38.60)	E (P/E: 41.35)
Fish meal	630	680	730	780	830
Fish oil	60	45	30	15	0
α-starch	120	100	80	60	40
Vitamin premix ^(1)^	20	20	20	20	20
Mineral premix ^(2)^	20	20	20	20	20
Sodium carboxymethyl cellulose	10	10	10	10	10
Microcrystalline cellulose	140	125	110	95	80
**Diet Compositions** (**% Dry Matter Basis**)					
Dry matter	97.97	97.61	97.61	97.26	97.42
Crude protein	38.66	41.90	44.78	48.04	51.71
Crude lipid	12.93	11.98	11.03	10.08	9.13
Ash	24.05	18.86	19.86	21.10	22.32
Gross energy (kJ/g)	18.78	18.75	18.46	18.18	18.07
Digestible energy (kJ/g) ^(3)^	12.77	12.69	12.61	12.53	12.45
Protein/energy (mg/kJ) ^(4)^	30.27	33.02	35.51	38.35	41.55

^(1)^ Vitamin premix (per kg of diet): vitamin A, 2000 IU; vitamin K3, 2.5 mg; vitamin B2 (riboflavin), 5 mg; vitamin B12, 0.025 mg; vitamin D3, 1200 IU; vitamin E, 21 mg; vitamin B1 (thiamin), 5 mg; vitamin B6, 5 mg; biotin, 0.05 mg; calcium pantothenic acid, 20 mg; nicotinamide, 25 mg; folic acid, 1.3 mg; inositol, 60 mg; ascorbic acid (35%), 110 mg. ^(2)^ Mineral premix (per kg of diet): FeSO_4_, 105 mg; Na_2_SeO_3_, 0.1 mg; KCl, 95 mg; NaCl, 165 mg; ZnSO_4_, 20 mg; KI, 1 mg; MnSO_4_, 10 mg; CuSO_4_, 12.5 mg; Co, 1.5 mg; MgSO_4_, 10 mg. ^(3)^ Digestible energy in kJ/g. ^(4)^ P/E: protein (mg)/digestible energy (kJ).

**Table 6:** Primers used in this study for quantitative real-time qPCR.

Gene	Primer Sequence (5′-3′)	Product Size (bp)	Annealing Temperature (°C)	Amplification Efficiency (%)
*AMPD1*	CATTTCCTTCCCGTGTT	242	58	103.6
	TCTGTCTGCGGAGTTGGT			
*GDH*	GACGACGACCCCAACTTCT	126	57	94.3
	GACCCGCTTCCTCTTCTGC			
*RPL13A*	CACCCTATGACAAGAGGAAGC	100	59	102.9
	TGTGCCAGACGCCCAAG			
*eEf2*	TCTGCTGTTATCCCGCCT	221	58	98.2
	TCGCCATCACTCCTCCTCT			
*LKB1*	GACGGGGCACTTAAAATC	136	58	98
	GTGTTACTCCAGCAGACCAAA			
*AMPK*	GGGATGCAAACCAAGATG	134	54	101.7
	ACAGACCCAGAGCGGAGA			
*mTOR*	GCATCAACGAGAGCACCA	113	55	96.5
	CGCTTCAAAATTCATAACCG			
*S6K*	CCTTCAAACCTTTCCTGCAATC	249	58	101.9
	ATTTAACTGGGCTGAGAGGTG			
*4EBP1*	ACTGACTGCCAGAAGACCA	167	58	100.8
	TTCTCATCGGCGTCCTT			
*NPY*	GTTGAAGGAAAGCACAGACA	202	52	98.2
	GCTCATAGAGGTAAAAGGGG			
*POMC*	GTGTCATCCTCGTTACTGC	268	58	100.3
	GCGACGCTCCTATTCAAT			
*AgRp*	GTGCTGCTCTGCTGTTGG	295	65	96.0
	AGGTGTCACAGGGGTCGC			
*CART*	CGAACCTAACCAGTGAGAAG	176	56	98.2
	GGGACAGTCGCACATCTT			
